# Mental health and use of Medicare Benefits Schedule follow-up mental health services by Indigenous people in Australia during the COVID-19 pandemic

**DOI:** 10.3389/fpubh.2023.1190484

**Published:** 2023-08-21

**Authors:** Kim Usher, Debra Jackson, Wenbo Peng, Suruchi Amarasena, Debbie McCowan, Joe Miller, Belinda Cashman, David Sibbritt

**Affiliations:** ^1^School of Health, Faculty of Medicine and Health, University of New England, Armidale, NSW, Australia; ^2^Susan Wakil School of Nursing and Midwifery, Faculty of Medicine and Health, The University of Sydney, Sydney, NSW, Australia; ^3^School of Public Health, Faculty of Health, University of Technology Sydney, Sydney, NSW, Australia; ^4^Walhallow Aboriginal Health Service, Quirindi, NSW, Australia; ^5^Armajun Health Service Aboriginal Corporation, Inverell, NSW, Australia; ^6^Aboriginal Maternal & Infant Health Service, Western Sydney Local Health District, Mount Druitt, NSW, Australia

**Keywords:** COVID-19 pandemic, Indigenous peoples, preventive health services, mental health, Medicare

## Abstract

**Background:**

Mental health care has declined during the COVID-19 pandemic as has attendance for preventive mental health health services. This study aimed to investigate trends in all types of mental health service claims identified in an Indigenous-specific health assessment for Indigenous people before and during COVID-19.

**Methods:**

We conducted an analysis of Medicare Benefits Scheme (MBS) mental health service items (Items 81,325 and 81,355), to investigate the trends in all types of mental health service claims specifically intended for Indigenous people of Australia. Data were analysed using descriptive statistics, including the total annual numbers of Indigenous peoples’ mental health service claims cross-tabulated by age groups and gender, between the calendar years 2017–2021. Multivariable Poisson regression modelling was used to determine associations that were statistically significant.

**Results:**

Our results indicate an overall rise in MBS claims for mental health follow-up services during 2019–2020 followed by a decline in 2020–2021. In addition, there was an overall decline in claims for follow-up psychology services across the time period 2019–2021.

**Conclusion:**

We found a significant decline in MBS items specific to follow-up mental health services (MBS Items 81,325 and 81,355) for Indigenous people in Australia suggesting a decline in attendance for mental health service follow-up which in turn may indicate a deficit in mental health care during the COVID-19 pandemic, an issue that may lead to poorer mental health outcomes in the future. Further research is needed to understand whether these changes were due to the impact of the COVID-19 pandemic or other factors.

## 1. Introduction

The COVID-19 pandemic is an unprecedented event in recent times. The impact the pandemic will have on global mental health, in both the short and longer term, remains unknown as the virus continues to plague us after three years. The COVID-19 pandemic has had a profound impact on healthcare across the globe (1). Strategies implemented to reduce the spread of the COVID-19 pandemic have had serious consequences for the health of many as a range of services were reduced or shut down during the pandemic to help restrict the spread of the virus. We know that strategies employed to help prevent the spread of the virus, led to serious economic, educational, social and mental health issues for both individuals and societies (2). It also resulted in many clinical appointments and elective surgeries being cancelled (3). In addition, many primary health services and general practitioner visits were reduced during the pandemic (4).

The COVID-19 virus was first detected in Australia in January 2020. In March 2020 there was a National lockdown followed by lockdowns related to following waves in 2020–2022. Fear of contracting the COVID-19 virus, meant many people were reluctant to attend health services or were unaware of health services that were available during the lockdowns (5). Failure to attend for preventive health services during the pandemic has been reported in a number of studies (6, 7).

There is evidence that mental health has declined across the globe since the onset of the recent COVID-19 pandemic (8, 9). This is not a new phenomenon as previous pandemics have resulted in similar outcomes demonstrating that not only does mental health decline during pandemics but that psychological distress can persist after the event (10, 11). A drop off in follow up mental health services may have long term consequences on mental health (12), especially for people with existing mental health conditions (13, 14). Certain groups of people may have been more likely to experience difficulty accessing services during the COVID-19 pandemic due to issues such as distance from services, or were reluctant to access services, including mental health services, during the pandemic because of previous negative experiences such as racism and discrimination (15, 16). This is the case for many Indigenous people in Australia who live in rural and remote areas and have higher rates of poor mental well-being than others in the population.

Medicare is a universal health insurance scheme that was funded by the Australian Commonwealth to provide free subsidised health professional services to Australians. The Medicare Benefits Schedule (MBS) is a key component of the Medicare system, including a range of consultation, diagnostic, and procedural/therapeutic services. MBS allocates a unique item number to each service (17). Some follow-up allied health services identified in an Indigenous-specific health assessment are specifically for patients of Indigenous descent after the Indigenous health assessment, with two of these services related to mental health (Items 81,325 and 81,355).

Indigenous people of Australia, similar to other Indigenous populations across the globe, experience worse health outcomes in general compared to the rest of the population. In 2018, the leading five causes of illness and death for Indigenous people in Australia were mental and substance use disorders, injuries, cardiovascular disease, cancer and musculoskeletal conditions (18). Preventative health care such as screening and treatment services, is however poorly accessed by Indigenous peoples in Australia despite their reported higher morbidity and mortality rate (19, 20), increased susceptibility to chronic illnesses and poorer health outcomes (21).

### 1.1. Aim

The aim of the study was to investigate trends in all types of mental health service claims identified in an Indigenous-specific health assessment for Indigenous people before and during COVID-19 by analysing MBS Items for mental health referrals for Indigenous people across all States and territories of Australia.

## 2. Methods

### 2.1. Study design and participants

A secondary data analysis was conducted on the publicly accessible data of MBS Items 81,325 and 81,355. These two items refer to Indigenous-specific MBS services, which are follow-up mental health services and psychological health services for Indigenous peoples who have had health assessments, respectively. Indigenous peoples who are eligible to claim those two MBS items were identified and referred by a medical practitioner as in need of such follow-up mental health services.

### 2.2. Procedures

The MBS data were downloaded from the Medicare Item Reports section of the Services Australia website from 1 January 2017 to 31 December 2021.

### 2.3. Sociodemographics

The dataset for each MBS item for Indigenous people across Australia was extracted and exported to Microsoft Excel for analysis, including the total numbers of mental health services used by age group, gender and calendar quarter.

### 2.4. Statistics

Data were analysed using descriptive statistics, including the total annual numbers of Aboriginal and Torres Strait Islander peoples’ mental health service claims cross-tabulated by age groups and gender, between the calendar years 2017–2021. Multivariable Poisson regression modelling was used to determine if the associations between the number of claims and age groups, gender, and calendar years were statistically significant.

## 3. Results

**Figure 1:**
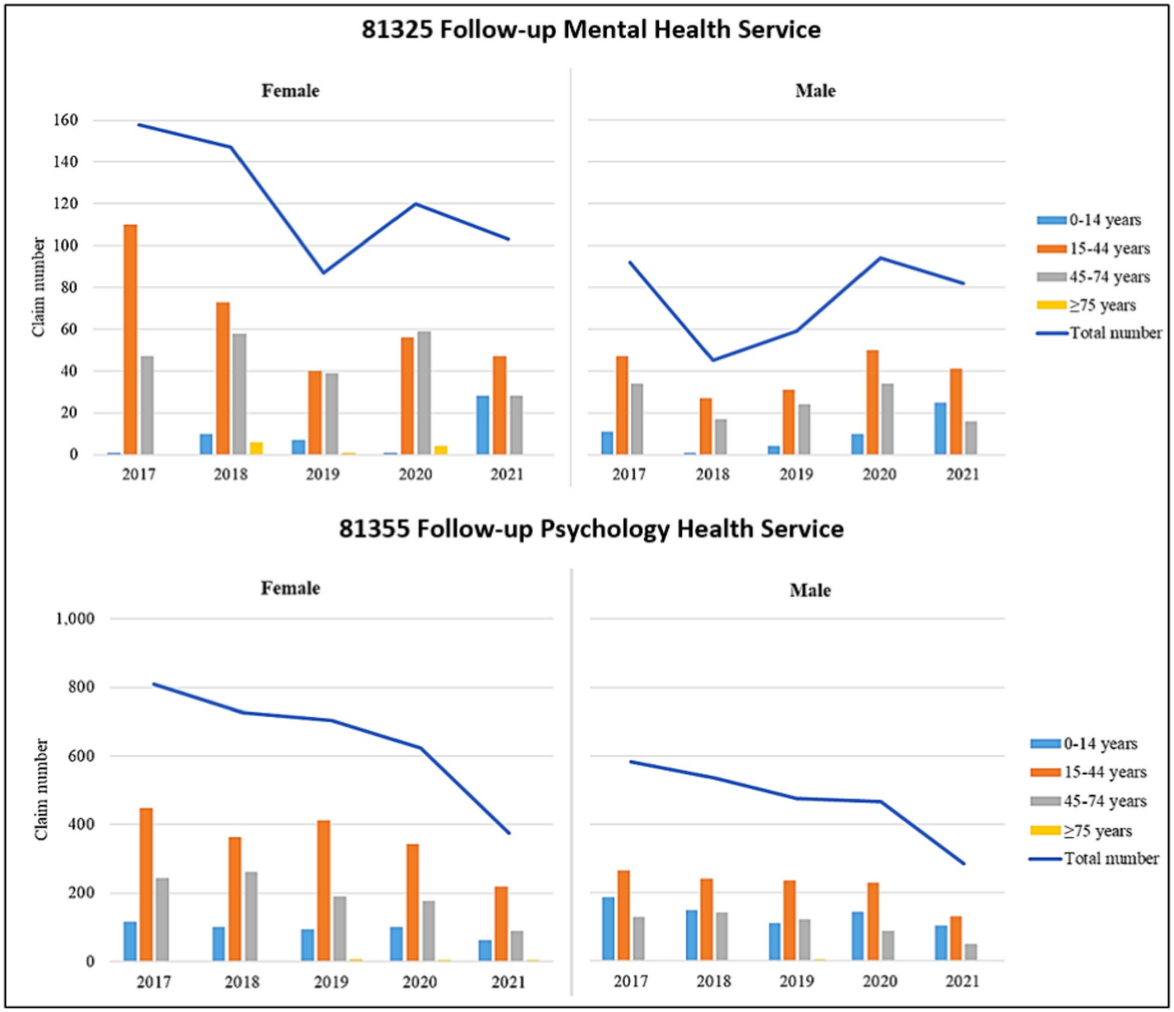
Australian Indigenous-specific mental health service claims, by age groups and gender, for the years 2017–2021. (1) there are different scales on the *y*-axis for the plots of the two different services and (2) analyses are based on services processed before 05/05/2022.

A total of 987 Indigenous-specific follow-up mental health services (Item 81,325) and 5,584 Indigenous-specific follow-up psychology health services (Item 81,355) were claimed from 1 January 2017 to 31 December 2021 by Indigenous people across Australia. As shown in Figure 1, for the Indigenous-specific follow-up mental health service claims referred by a medical practitioner, there was a reduction of 41.6% in the annual total claim numbers between 2017 (*n* = 250) and 2019 (*n* = 146). This was followed by a considerable increase of 46.6% in the annual total claim number in 2020 (*n* = 214) and a slight decline in 2021 (*n* = 185). In comparison, there was a decline of 21.8% in the annual total claim numbers of Indigenous-specific follow-up psychology health services (Item 81,355) over time from 2017 (*n* = 1,393) to 2020 (*n* = 1,090). This was followed by a considerable decline of 39.4% to 2021 (*n* = 660).

Figure 1 presents the annual claim numbers of Indigenous-specific mental health and psychological health services referred by a medical practitioner by age group and gender, for the years 2017–2021. For mental health service claims (Item 81,325), there was a similar trend for males and females, in that there was an increase in the number of service claims from 2019 to 2020, followed by a decrease in 2021. However, males claimed much fewer mental health services over the period compared to females. Also, regardless of gender, the 15–44 years age group had the greatest number of claims across most years, followed by the 45–74 years age group except for 2021 when there was a considerable increase in claims by children in the 0–14 years age group.

The claim trend for Indigenous people seeking follow-up psychological health services (Item 81,355) was consistent for both males and females, showing a gradual decline from 2017 to 2020, and then a steeper decline in 2021. Further, females claimed more psychological health services than males each year. Additionally, the 15–44 years age group had the greatest number of claims across all years regardless of gender, while the 45–74 years age group and 0–14 years age group (except for 2019) had the second highest number of claims across all years among females and males, respectively (Figure 1).

Table 1 shows the output from the Poisson regression models. For Indigenous-specific mental health (Item 81,325) and psychological health services (Item 81,355) referred by a medical practitioner, the number of claims was significantly associated with not only gender and age group but also calendar years. Specifically, for mental health services, compared to 2017, all following years had significantly lower numbers of claims, with the largest differences occurring in 2020 and 2021. That is, after adjusting for gender and age group, there were 22% (RR = 0.78; 95% C.I.: 0.72, 0.85; *p* < 0.001) less claims in 2020 and 53% (RR = 0.47; 95% C.I.: 0.43, 0.52; *p* < 0.001) less claims in 2021. Similarly, compared to 2017, all following years apart from 2020 had significantly lower numbers of psychological health service claims, with the largest differences occurring in 2019 and 2021. That is, after adjusting for gender and age group, there were 42% (RR = 0.58; 95% C.I.: 0.48, 0.72; *p* < 0.001) less claims in 2019 and 26% (RR = 0.74; 95% C.I.: 0.61, 0.89; *p* < 0.001) less claims in 2021.

**Table 1:** The associations between the claim number of Australian Indigenous-specific mental health services and gender, age group and year.

Factor		Risk ratio	95% C.I.	*p*-value
Follow-up mental health service (Item 81,325)				
Gender	Female	1.0	–	–
	Male	0.72	0.69, 0.76	<0.001
Age group (years)	0-14	1.0	–	
	15-44	2.47	2.31, 2.64	<0.001
	45-74	1.28	1.18, 1.38	<0.001
	75+	0.03	0.02, 0.04	<0.001
Calendar year	2017	1.0	–	
	2018	0.91	0.84, 0.98	0.011
	2019	0.85	0.78, 0.91	<0.001
	2020	0.78	0.72, 0.85	<0.001
	2021	0.47	0.43, 0.52	<0.001
Follow-up psychological health service (Item 81,355)				
Gender	Female	1.0	–	
	Male	0.61	0.53, 0.69	<0.001
Age group (years)	0-14	1.0	–	
	15-44	5.22	4.21, 6.47	<0.001
	45-74	3.56	2.85, 4.44	<0.001
	75+	0.11	0.06, 0.21	<0.001
Calendar year	2017	1.0	–	
	2018	0.77	0.64, 0.93	0.006
	2019	0.58	0.48, 0.72	<0.001
	2020	0.86	0.72, 1.04	0.116
	2021	0.74	0.61, 0.89	0.002

Risk ratio obtained from multivariable poisson regression model. 95% C.I. refers to 95% confidence interval.

## 4. Discussion

We undertook a secondary analysis of publicly available data to determine whether the COVID-19 pandemic had influenced the number of MBS claims for mental health referral services for Indigenous Australians. There was an increase in the number of claims from 2019 to 2020 for mental health services, so the COVID-19 pandemic may have had an impact during that time (i.e., increase in mental health issues led to increase in referrals). Hence, this may explain the rise in claims for follow-up mental health services early in the COVID-19 pandemic. There was however a decline in claims from 2020 to 2021; this may indicate a reluctance to attend for follow-up mental health services related to the COVID-19 pandemic or could be the result of some other unknown factor (access issues or other reasons). These findings are not dissimilar to those of other researchers. There is evidence that mental health issues have escalated across the world since the onset of the COVID-19 pandemic (8, 9, 22). As a result, it is reasonable to surmise that there has also been an increased need for follow-up mental health services for Indigenous people since the onset of the COVID-19 pandemic. However, there is also evidence that people have been reluctant to attend for healthcare services, particularly preventive health services, due to the COVID-19 pandemic (6, 7). In addition, many medical services were severely disrupted during the COVID-19 pandemic (3, 4) so it is impossible to rule out the impact of that situation on the reduction of item claims for services relevant to Indigenous people. It is thus possible that the COVID-19 pandemic has led to a decrease in referral for follow-up services due to a reduction in health assessments by general practitioners, or that Indigenous people have made a concious decision not to attend for health assessments where they might be referred for follow-up treatment, or that services were not available. In any case, a hypothesised reduction of attendance for follow-up mental health services is a concern given the evidence for esclating mental health issues during the COVID-19 pandemic.

Interestingly, claims for psychology health services declined throughout the COVID pandemic period, but there was a steeper drop from 2020 to 2021, suggesting the COVID pandemic may have had an impact (or there might just be a lack of demand for these services over time). This finding seems to be contradictory to other previous evidence that need for services rose in the beginning of the pandemic. Exactly why Indigenous people did not attend for psychological services or were not referred for psychological services is unclear. The drop in psychology item claims may indicate the alternate use of telehealth services (not identified in this study), but telehealth services for mental health were not available at the commencement of, or prior to the commencement of the COVID-19 pandemic. Mental health services delivered by telehealth peaked in September 2021, and by January 2022, 30% of MBS mental health services were delivered via telehealth (18). Telehealth use has grown among Indigenous populations since the COVID-19 pandemic began. However, there are still considerable barriers to its use including privacy and confidentiality, internet availability, as well as low health and digital literacy (23).

Age and gender also had an impact on MBS mental health claims with claims for males less than those for females. Research conducted in Australia during the COVID-19 pandemic has reported women and younger people across the population experiencing higher levels of psychological distress due to the COVID-19 pandemic (18, 24). The age group 15–44 years had the highest number of claims across most of the years (24). There is evidence to indicate that the mental health of young people in the adolescent range (15–24 year olds) has significantly deteriorated internationally since the development of the COVID-19 pandemic (25). Australian Indigenous young people are considered to be at greater risk of negative mental health outcomes in many cases (26).

## 5. Limitations

Our study has some limitations. Firstly, this study only uses a secondary dataset for analyses. Therefore, individual-level MBS data are not available in the publicly accessible MBS database. With this data, we can only consider associations between claim trends and the impact of the COVID pandemic, rather than causation. Second, MBS data used for analysis only included services provided by health professionals who are registered with the Department of Human Services. Mental health services provided by hospital doctors and services that qualify for a benefit under the Department of Veterans Affairs were not available. Our research findings thus may not be generalized to all Indigenous people in Australia to understand their mental health needs during the pandemic. Third, this paper excluded MBS item numbers available for both the general population and Indigenous people. Thus, the claim number of Indigenous-specific follow-up mental health services referred by a medical practitioner may be lower than the true value. Last, the lack of awareness among Indigenous Australians regarding the accessibility and availability of these mental health services during the COVID-19 pandemic might have caused the drop or decline in follow-up mental health services.

## 6. Conclusion

In this study we found a significant decline in MBS items specific to follow up mental health services (MBS Items 81,325 and 81,355) for Indigenous people in Australia during the COVID-19 pandemic indicating a reduction in attendance. These findings suggest the COVID-19 pandemic may be associated with a decline in attendance for mental health service follow-up which in turn may indicate a deficit in mental health care during COVID-19 pandemic, an issue that may lead to poorer mental health outcomes in the future. Given the importance of accessing mental health services for those in need, health services need to pro-actively and collaboratively work with Indigenous peoples of Australia to develop strategies to overcome this issue in future pandemics.

## Data availability statement

Publicly available datasets were analyzed in this study. This data can be found here: the datasets generated and analysed during the current study are publicly available in the Medicare Item Reports provided by Services Australia via http://medicarestatistics.humanservices.gov.au/statistics/mbs_item.jsp.

## Ethics statement

Ethical approval was not required for the study involving humans in accordance with the local legislation and institutional requirements. Written informed consent to participate in this study was not required from the participants or the participants’ legal guardians/next of kin in accordance with the national legislation and the institutional requirements.

## Author contributions

KU, DJ, DS, and WP conceived and designed the study. DS and WP undertook the data retrieval and analysis. SA provided clinical advice the data analysis. KU wrote the first draft of the paper. All authors contributed to the article and approved the submitted version.

## Funding

The project was funded by a NSW Department of Health COVID-19 grant (round 1).

## Conflict of interest

The authors declare that the research was conducted in the absence of any commercial or financial relationships that could be construed as a potential conflict of interest.

The reviewer BH declared a shared affiliation with the author DJ to the handling editor at the time of review.

## Publisher’s note

All claims expressed in this article are solely those of the authors and do not necessarily represent those of their affiliated organizations, or those of the publisher, the editors and the reviewers. Any product that may be evaluated in this article, or claim that may be made by its manufacturer, is not guaranteed or endorsed by the publisher.
